# Significant Impact of the Ketogenic Diet on Low-Density Lipoprotein Cholesterol Levels

**DOI:** 10.7759/cureus.9418

**Published:** 2020-07-27

**Authors:** Jesus Salas Noain, Arun Minupuri, Ajinkya Kulkarni, Shengnan Zheng

**Affiliations:** 1 Internal Medicine, Mercy Catholic Medical Center, Darby, USA

**Keywords:** ldl-c, lipid metabolism, low carb diet, risk factors cardiovascular diseases, low-carb diet

## Abstract

It is well known, based on the previous research, that a ketogenic diet leads to an improvement in the lipid profile and decreases cardiovascular risk factors such as hypertension. However, recent studies have also reported increased levels of total cholesterol and low-density lipoprotein cholesterol (LDL-C) as a result of this diet. It has been postulated that this elevation in LDL-C would not likely increase cardiovascular complications due to the large LDL-C particle size. In this case report, we present a case of a rapid increase, followed by a rapid correction of LDL-C, in a patient following a ketogenic diet.

A 56-year-old Hispanic female with a past medical history of hypertension and fibromyalgia presented to the outpatient clinic for evaluation of fatigue. She reported that she had been following a strict ketogenic diet along with daily regular exercise for approximately 30-40 days prior to this visit. Her diet consisted of low-carbohydrate vegetables, seafood, avocados, eggs, and coconut oil. The patient’s physical exam was unremarkable. At the time of the visit, her BMI was calculated at 28 kg/m^2^, with a weight loss of approximately six to seven pounds since starting the ketogenic diet. Her fasting lipid profile showed a total cholesterol of 283 mg/dl, LDL-C of 199 mg/dl, high-density lipoprotein cholesterol (HDL-C) of 59 mg/dl, and triglycerides levels of 124 mg/dl. She was instructed to stop the ketogenic diet and to incorporate a balanced diet, which includes a higher amount of carbohydrates and lower fat. She was also started on high-intensity atorvastatin. However, she reported experiencing myalgias soon after initiating atorvastatin; therefore, the medication was switched to rosuvastatin 10 mg at bedtime. During her follow-up appointment, she reported not having consistently taken rosuvastatin due to the concern of worsening myalgias. Her lipid profile, after four weeks of ketogenic diet discontinuation and inconsistent use of statins, showed significant improvement resulting in a total cholesterol level of 190 mg/dl and LDL-C of 106 mg/dl. Statin therapy was discontinued, and the patient maintained optimal LDL-C levels on subsequent testing.

This patient showed a rapid increase in LDL-C and total cholesterol after only 30-40 days of the ketogenic diet. Her drastic elevation in LDL-C could also be explained due to the rapid weight loss, as cholesterol in the adipose tissue is known to mobilize as the fat cells shrink. Interestingly, her BMI four weeks after the discontinuation of the ketogenic diet did not change despite a marked improvement in her LDL-C. Therefore, we believe the acute onset and resolution of hyperlipidemia was secondary to the ketogenic diet itself. This study helps to better understand expectations when recommending a ketogenic diet to patients and its consequences. There is currently no statistically significant study that proves this elevation of LDL-C would not increase cardiovascular risks. Furthermore, the necessity for statin therapy in a ketogenic diet-induced hyperlipidemia remains unknown.

## Introduction

Recently, ketogenic diet has gained significant popularity due to studies showing possible cardiovascular benefits [1]. This diet is quite unorthodox compared to other diets as it includes food with a very low-carbohydrate and high-fat content that aims to drastically reduce carbohydrate intake and replace it with fat, hence inducing ketosis. A typical ketogenic diet usually includes coconut oil, butter, eggs, avocados, cheese, and meat. Ketogenic diet has been shown to be effective in treating cases of obesity, diabetes type 2 [1], and metabolic syndrome [2]. Occasionally, this diet is recommended for uncontrolled epilepsy due to the brain-protecting effect from ketosis, resulting in fewer seizure episodes [3]. Previous studies reported a beneficial effect with a short-term ketogenic diet, as well as a long-term ketogenic diet for reducing body weight and body mass index in patients [4]. Furthermore, it was also found that the diet decreases levels of triglycerides and blood glucose, and increases levels of high-density lipoprotein cholesterol (HDL-C) [5].

Whereas carbohydrate restriction and ketosis induction improve dyslipidemia, the effects on total cholesterol and low-density lipoprotein cholesterol (LDL-C) are less predictable [6]. Usually LDL-C and total cholesterol remain near baseline; however, an increase in LDL-C levels has also been reported in some patients [7]. It has been postulated that this elevation in LDL-C would likely not increase cardiovascular complications due to its larger particle size [8]. In this case report, we present a case of a rapid increase, followed by a rapid correction of LDL-C in a patient following a ketogenic diet.

## Case presentation

A 56-year-old Hispanic female with a past medical history of hypertension and fibromyalgia presented to the outpatient clinic for the evaluation of fatigue. She reported that she had been following a strict ketogenic diet along with daily regular exercise for approximately 30-40 days prior to this visit. Her diet consisted of low-carbohydrate vegetables, seafood, avocados, eggs, and coconut oil. The patient’s physical examination was unremarkable. At the time of the visit, her BMI was calculated at 28 kg/m^2^, with a weight loss of approximately six to seven pounds since starting the ketogenic diet. Her fasting lipid profile showed a total cholesterol level of 283 mg/dl, LDL-C of 199 mg/dl, HDL-C of 59 mg/dl, and triglyceride level of 124 mg/dl. Her creatine phosphokinase (CPK) level was 94 U/l. She was instructed to stop the ketogenic diet and to incorporate a balanced diet, which includes a higher amount of carbohydrates and lower fat. She was also started on high-intensity atorvastatin. However, she reported experiencing myalgias soon after initiating atorvastatin; therefore, the medication was switched to rosuvastatin 10 mg at bedtime. During her follow-up appointment, she reported not having consistently taken rosuvastatin due to concerns of worsening myalgias. CPK levels were reordered and found to be unchanged. Furthermore, she denied prominent muscle pain, tenderness, or weakness during this follow-up appointment; hence, statin-induced myopathy was ruled out. Her lipid profile, after four weeks of ketogenic diet discontinuation and inconsistent use of statins, showed a significant improvement resulting in a total cholesterol level of 190 mg/dl and LDL-C of 106 mg/dl. Statin therapy was discontinued, and the patient maintained optimal LDL-C levels on subsequent testing.

## Discussion

This patient showed a rapid increase in LDL-C and total cholesterol after only 30-40 days of the ketogenic diet. The drastic elevation in her LDL-C could also be explained due to the rapid weight loss, as cholesterol in the adipose tissue is known to mobilize as the fat cells shrink [9]. Interestingly, her BMI four weeks after the discontinuation of ketogenic diet did not change despite a marked improvement in her LDL-C. Therefore, we believe the acute onset and resolution of hyperlipidemia was secondary to the ketogenic diet itself. Unfortunately, she was unable to tolerate statin therapy due to myalgia. This was likely secondary to fibromyalgia, rather than statin-induced myopathy, as CPK levels were within normal limits.

The dietary cholesterol intake increases by >100% when patients are switched to a standard ketogenic diet, which leads to increases in total cholesterol and LDL-C. Interestingly, in some studies, these two biomarkers were not significantly elevated after six weeks of the ketogenic diet [5]. There are also changes in the LDL subfractions, which are thought to be beneficial for cardiovascular disease prevention. However, in some cases, the predominantly LDL subfractions are small, dense LDL particles, which are known to increase the risk of cardiovascular disease. Therefore, close routine monitoring of body weight, lipid panel including LDL subfractions, and glucose levels should be strongly considered in patients following a ketogenic diet. Furthermore, due to the significant variability of LDL-C levels and LDL subfraction predominance type, avoiding the ketogenic diet in patients with significant cardiovascular disease should be discussed with the patients. An alternative for these patients is a Mediterranean diet, which has been shown to decrease the risk of cardiovascular disease and improve dyslipidemia in different studies [10].

## Conclusions

This study helps to better understand expectations when recommending a ketogenic diet to patients and its consequences. There is currently no statistically significant study that proves this elevation in LDL-C would not increase cardiovascular risks. Furthermore, the necessity for statin therapy in a ketogenic diet-induced hyperlipidemia remains unknown. It is also unknown if age and concomitant exercise while following a ketogenic diet play a role in the fluctuating lipid levels. Due to the unpredictable response of LDL-C levels to a ketogenic diet, close monitoring of patients with a high risk of cardiovascular disease should be considered. Further investigations on fluctuating lipid, BMI, CPK levels and cardiovascular risks are needed.

## References

[1] Feinman RD, Pogozelski WK, Astrup A (2015). Dietary carbohydrate restriction as the first approach in diabetes management: critical review and evidence base. Nutrition.

[2] Sackner-Bernstein J, Kanter D, Kaul S (2015). Dietary intervention for overweight and obese adults: comparison of low-carbohydrate and low-fat diets. A meta-analysis. PLoS One.

[3] El-Rashidy OF, Nassar MF, Abdel-Hamid IA (2013). Modified Atkins diet vs classic ketogenic formula in intractable epilepsy. Acta Neurol Scand.

[4] Volek JS, Fernandez ML, Feinman RD, Phinney SD (2008). Dietary carbohydrate restriction induces a unique metabolic state positively affecting atherogenic dyslipidemia, fatty acid partitioning, and metabolic syndrome. Prog Lipid Res.

[5] Sharman MJ, Kraemer WJ, Love DM, Avery NG, Gómez AL, Scheett TP, Volek JS (2002). A ketogenic diet favorably affects serum biomarkers for cardiovascular disease in normal-weight men. J Nutr.

[6] Volek JS, Sharman MJ, Gómez AL, Scheett TP, Kraemer WJ (2003). An isoenergetic very low carbohydrate diet improves serum HDL cholesterol and triacylglycerol concentrations, the total cholesterol to HDL cholesterol ratio and postprandial pipemic responses compared with a low fat diet in normal weight, normolipidemic women. J Nutr.

[7] Friedewald WT, Levy RI, Fredrickson DS (1972). Estimation of the concentration of low-density lipoprotein cholesterol in plasma, without use of the preparative ultracentrifuge. Clin Chem.

[8] Hoogeveen RC, Gaubatz JW, Sun W (2014). Small dense low-density lipoprotein-cholesterol concentrations predict risk for coronary heart disease: the Atherosclerosis Risk in Communities (ARIC) study. Arterioscler Thromb Vasc Biol.

[9] Blazek A, Rutsky J, Osei K, Maiseyeu A, Rajagopalan S (2013). Exercise-mediated changes in high-density lipoprotein: impact on form and function. Am Heart J.

[10] Martinez-Gonzalez MA, Gea A, Ruiz-Canela M (2019). The Mediterranean diet and cardiovascular health. Circ Res.

